# Solubilization and Humanization of Paraoxonase-1

**DOI:** 10.1155/2012/610937

**Published:** 2012-06-07

**Authors:** Mohosin Sarkar, Christina Keventzidis Harsch, George T. Matic, Kathryn Hoffman, Joseph R. Norris, Tamara C. Otto, David E. Lenz, Douglas M. Cerasoli, Thomas J. Magliery

**Affiliations:** ^1^Department of Chemistry, The Ohio State University, Columbus, OH 43210, USA; ^2^Physiology and Immunology Branch, Research Division, United States Army Medical Research Institute of Chemical Defense, Aberdeen Proving Ground, MD 21010, USA; ^3^Department of Biochemistry, The Ohio State University, Columbus, OH 43210, USA

## Abstract

Paraoxonase-1 (PON1) is a serum protein, the activity of which is related to susceptibility to cardiovascular disease and intoxication by organophosphorus (OP) compounds. It may also be involved in innate immunity, and it is a possible lead molecule in the development of a catalytic bioscavenger of OP pesticides and nerve agents. Human PON1 expressed in *E. coli* is mostly found in the insoluble fraction, which motivated the engineering of soluble variants, such as G2E6, with more than 50 mutations from huPON1. We examined the effect on the solubility, activity, and stability of three sets of mutations designed to solubilize huPON1 with fewer overall changes: deletion of the N-terminal leader, polar mutations in the putative HDL binding site, and selection of the subset of residues that became more polar in going from huPON1 to G2E6. All three sets of mutations increase the solubility of huPON1; the HDL-binding mutant has the largest effect on solubility, but it also decreases the activity and stability the most. Based on the G2E6 polar mutations, we “humanized” an engineered variant of PON1 with high activity against cyclosarin (GF) and found that it was still very active against GF with much greater similarity to the human sequence.

## 1. Introduction

Paraoxonase-1 (PON1) is a mammalian serum protein, the activity of which is related to cardiovascular health and the toxicology of organophosphorus (OP) compounds [1–3]. PON1 is thought to be synthesized mostly in the liver, and it is associated with high-density lipoproteins (HDLs) in serum [4]. The exact function of PON1 is not known, but it is an efficient hydrolase of lactones and esters and an inefficient hydrolase of OP compounds, including pesticide metabolites such as paraoxon (from parathion) and chlorpyrifos oxon, and nerve agents such as sarin, tabun, and VX [1, 5]. Increased PON1 activity appears to be related to lower levels of oxidation of low-density lipoprotein (LDL) particles, and its hydrolytic activity has been suggested to be directed at oxidized fatty acids and homocysteine thiolactone [6–8]. Its increased activity has been shown to be related to decreased atherosclerosis, and it has been implicated in mechanisms of cholesterol efflux [9, 10]. PON1 also efficiently hydrolyzes bacterial lactones involved in quorum sensing, and it may contribute to innate immunity through this activity [11]. Although the hydrolysis of OP compounds is almost certainly a promiscuous activity of the enzyme, it contributes to the susceptibility to OP intoxication [12], and PON1 has been suggested as a lead molecule for a prophylactic or therapeutic bioscavenger of OP toxins [13, 14]. Human PON1, particularly the R192 alloform, is already sufficiently active to protect against chlorpyrifos oxon and diazoxon exposures without engineering. The turnover of many other OPs by natural PON1 is not sufficient to afford significant protection, but a mammalian chimeric form of PON1 has recently been engineered for significant activity against some G-agents [15].

**Figure 1:**
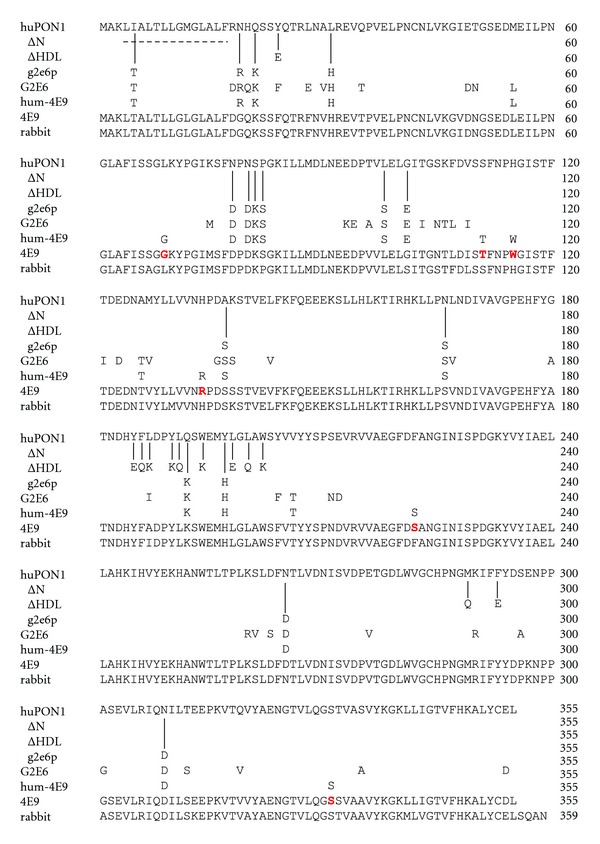
*Alignment of PON1 variants.* All differences are noted with respect to the human PON1 sequences (Q192/M55 polymorph); the 4E9 sequence and the similar rabbit PON1 sequence are shown in full for reference. Differences between G3C9 and 4E9 are noted in red in the 4E9 sequence. -: deletion.

As a result of the physiological and toxicological correlations with increased PON1 activity, there is great motivation to develop PON1 as a therapeutic agent. There are significant difficulties with this: PON1 has only moderate solubility; it has three Cys residues including two forming a disulfide bond, and it is glycosylated [16]. Human PON1 (huPON1) is very difficult to produce in soluble, folded form in *E. coli*. Large-scale fermentation has been used to produce soluble huPON1 successfully in *E. coli*, but in poor yields for pharmaceutical production [14]. This motivated Aharoni and colleagues to generate a chimeric mammalian PON1 by DNA shuffling of mouse, rat, rabbit, and human PON1 isoforms, resulting in a variant called G2E6 that could be expressed when fused to the C-terminus of thioredoxin in good yields in the soluble fraction of *E. coli* (Figure 1) [17]. The crystal structure of G2E6 was solved, revealing it to be a six-bladed *β*-propeller protein bound to two Ca^2+^ ions, one of which appears to play a more structural role and one of which is located in what is presumed to be the active-site pocket [16]. A further generation of DNA shuffling and selection yielded G3C9 PON1, which can be expressed in significant amounts in the soluble fraction of *E. coli *without a fusion partner (although it bears a C-terminal hexahistidine tag). Both of the proteins have greatest sequence similarity to the rabbit isoform of PON1, and they differ by 58-59 (G2E6) and 50-51 (G3C9) amino acids from huPON1, depending on the polymorph, mostly on the surface and essentially not at all in the putative active site. (Human PON1 is either Leu or Met at 55, while rabbit is only known to be Leu at this position).

 The poor solubility of human PON1 is presumably in part a consequence of its ability to associate with HDL. The nature of this interaction is not clearly defined. PON1 has a signal sequence directing it for cellular export, and it is mutated at the cleavage site for the signal protease, resulting of retention of the hydrophobic signal peptide [18, 19]. That peptide is disordered in the structure of G2E6 [16]. There is a large hydrophobic patch on the surface of PON1 that is near the N-terminus, suggesting that this is the HDL interaction surface. The interaction of PON1 with HDL stimulates its activity towards lactones, and removal of the signal peptide (residues 1–20) has been shown to abrogate that stimulation, suggesting that it is critical for proper embedding in the apoA-I HDL particle [20]. It is not surprising that most of the differences between the very insoluble human PON1 and the more soluble G2E6 and G3C9 are on the surface of the protein, as this is where changes would be most expected to affect the solubility of the folded protein.

 Despite this intuitive expectation, surprisingly little is known about how mutations affect the solubility of proteins or how to engineer proteins for greater solubility. Several studies have reengineered membrane proteins to render them soluble. Li and coworkers reengineered phospholamban (PLB), a protein that forms a stable helical homopentamer within the sarcoplasmic reticulum membrane, into a soluble pentameric helical bundle by replacing its lipid-exposed hydrophobic residues with charged and polar residues [21]. Based on computational design, Slovic and coworkers rationally engineered a water-soluble analog of PLB by changing membrane-exposed positions to polar or charged amino acids, while the putative core was left unaltered [22]. These constructs were based on the hypothesis that membrane proteins and water-soluble proteins share a similar core and it should be possible to solubilize membrane proteins by mutating only their lipid-exposed residues. The redesigned PLBs mimic all of the reported properties of PLB including oligomeric state, helical structure, and stabilization upon phosphorylation. Based on the same approach, Slovic and coworkers redesigned a water-soluble variant of a membrane protein, potassium channel KcsA, by mutating the lipid-contacting side chains to more polar groups [23].

 We were interested in determining how mutations to huPON1 would affect its solubility and soluble expression in *E. coli*. We hypothesized that three different types of mutations might increase the solubility of human PON1. We speculated that (a) removal of the hydrophobic N-terminal leader sequence and (b) mutations of hydrophobic amino acids in the presumptive HDL binding site to polar residues would increase the solubility. We also speculated that (c) the surface residues which were mutated to be more polar amino acids during the directed evolution of G2E6 PON1 were mostly responsible for the increased solubility. To test these ideas, we constructed three mutants of human PON1 called ΔN-huPON1, ΔHDL-huPON1, and g2e6p-huPON1 (Figure 1). We also combined some of the mutations to look for additive effects on solubility.

 To test the solubility of these proteins, we exploited the screen developed by Waldo and colleagues based on fusion of an analyte protein (“protein of interest” or POI) to the N-terminus of the “folding reporter” variant of green fluorescent protein (frGFP) [24]. Briefly, if the POI folds and is soluble, then the frGFP also folds and its chromophore develops, resulting in fluorescent cells. If the POI is insoluble, then the fusion is found in the membrane-associated fraction and little fluorescence develops. Waldo and colleagues demonstrated that the amount of cellular fluorescence is related to the amount of soluble protein. Consequently, we fused each of the huPON1 variants to the N-terminus of frGFP and determined the fluorescence level of the host bacterial cells. We also examined the activity and stability of the resulting proteins.

 We were also interested in the determinants of huPON1 solubility because we wished to use that knowledge to generate variants of engineered PON1 that had significantly greater activity toward OP agents, but with a surface sequence significantly more like native huPON1. Little is known about the immunological effects of the administration of heterologous variants, but the large number of mutations on the surface of G2E6 and G3C9 relative to human PON1 is a cause for concern. In the field of antibody-based therapeutics, human anti-mouse antibody syndrome is a common effect of the administration of mouse-derived antibodies, and so variants of mouse antibodies have been successfully “humanized” by replacing their surface residues with a human sequence while maintaining the binding site residues elicited during affinity maturation [25]. We speculated that we might be able to “humanize” or at least partially humanize evolved variants of G3C9 PON1 with high OP activity by reverting the surface back to the human sequence, except for solubilizing mutations identified in the first part of this work. We chose to humanize the recently reported 4E9 variant (Figure 1) [15], which has very high activity against the cyclosarin (GF) analog CMP, using the same strategy used to generate g2e6p-huPON1. Because the substrate specificities of huPON1 and G2E6 PON1 are quite different despite essentially identical active sites, it was not clear if humanization could yield an active enzyme [5]. Here we show that humanization of 4E9 was successful and suggests a path forward for improved therapeutics based on engineered PON1 variants.

## 2. Materials and Methods

### 2.1. Cloning huPON1 and frGFP Fusions in pET11a

**Figure 2:**
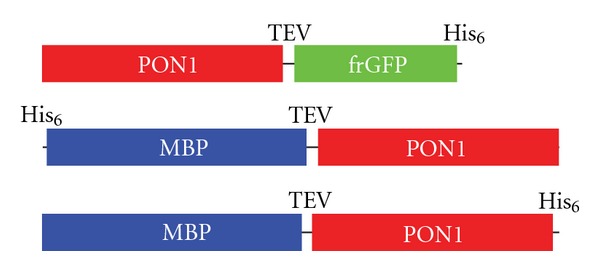
*Schematics of the fusions used in this study.* PON1, paraoxonase-1 variant; TEV, TEV protease site; frGFP, folding reporter GFP; His_6_, hexahistidine tag; MBP, maltose-binding protein.

The frGFP gene was generated in our lab from the genes for GFPuv [26] and EGFP [27] by overlap PCR, resulting in a GFP with mutations F64L S65T F99S M153T V163A. The frGFP gene was PCR amplified with primers coding for a 6 × His tag and an AatII site at the 3′ end and an EcoRI site at the 5′ end. Wild-type human PON1 (Q192/M55) was PCR amplified from a mammalian expression vector, pcDNA3. The oligonucleotide (Sigma Genosys, The Woodlands, TX) primers 5′-AATAATTATC ATATGGCTAA GCTGATTGCG CTCACCC-3′ with an NdeI site, and 5′-ATAATGAATT CGCCGCTGCT TCCGCTCTGA AAATACAGAT TCTCACCGCC GGTACCGAGT TCGCAGTAAA GAGCTTTGTG AAACAC-3′ coding for a KpnI site, TEV protease (ENLYFQG) site, linker (GSSG) and EcoRI site, were used for amplification. A fusion of huPON1-(KpnI-)TEV-Linker-(EcoRI-)frGFP (Figure 2) was cloned into a pET11a vector between the NdeI and AatII sites using a three-piece ligation. For reference, the G2E6 gene was analogously cloned into this construct (the gene was kindly provided by Dan Tawfik, Weizmann Institute of Science). The sequence of the fusion construct was confirmed by DNA sequencing (Genewiz, South Plainfield, NJ).

### 2.2. Rationally Engineered huPON1 Variants

**Figure 3:**
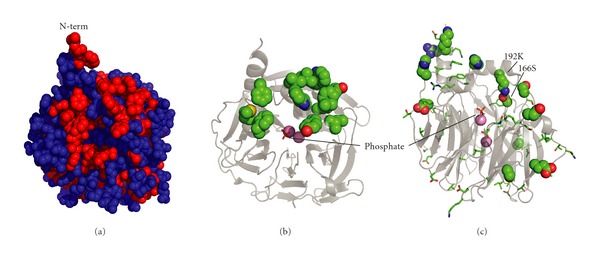
*PON1 solubilizing mutations.* (a) The surface of G2E6 is shown, with hydrophobic amino acids (VGMCILYFW) shown in red. Residues 1–15 are not resolved in the X-ray crystal structure, but the ΔN-huPON1 variant removed residues 4–17. (b) The positions modified in the ΔHDL-huPON1 variant are shown in spheres. These residues compose much of the hydrophobic surface patch near the N-terminus evident in (a). The Ca^2+^ ions are shown as pink spheres, and a phosphate bound in the presumed active site is shown in orange sticks. (c) The 59 positions that differ between huPON1 and G2E6 are shown; the positions that were modified in g2e6p-huPON1 are spheres, and the other 43 positions are sticks. Position 166, which was modified because of its proximity of 192, is noted. Rendered from PDB ID: 1V04 with PyMOL.

 To generate ΔHDL-huPON1 (Figures 1 and 3), twelve Glu, Gln, or Lys mutations were made at hydrophobic residues in the putative HDL binding site (Y24E, Y185E, F186Q, L187K, Y190K, L191Q, W194K, L198E, L200Q, W202K, M289Q, F293E) in the huPON1 construct. All of these mutations were introduced at once by overlap PCR. Three pairs of oligonucleotides were used to amplify three fragments of the huPON1 gene with all 12 mutations. The three fragments were then subjected to assembly and amplification similar to the final steps of DNA shuffling using two terminal primers, 5′-ATAGATATAC ATATGGCGAA GCTGATTGCA CTCACGCTCT TGGGGATGGG ACTGGC ACTC TTCAGGAACC ACC-3′ and 5′-CTCACCGCCG GTACCGAGTT CGCAGTAAAG AGCTTTG-3′. These primers coded for NdeI and KpnI sites. The amplified ΔHDL-huPON1 gene was cloned into pET11-huPON1-TEV-frGFP vector, replacing huPON1 between NdeI and KpnI, and the construct was confirmed by DNA sequencing.

 The g2e6p-huPON1 was engineered by introducing surface mutations that became more polar in the directed evolution of G2E6 from huPON1. Fifteen such mutations (I5T, N19R, Q21K, L31H, N78D, N80D, S81K, P82S, L98S, G101E, A137S, Q192K, Y197H, N265D, and N309D) and an N166S compensatory mutation of the Q192K mutation were introduced into huPON1 by total gene synthesis using the TBIO method [28]. Thirty primers of 60 nt coding for all 16 point mutations were designed using DNAWorks [29]. The assembled full-length gene for g2e6p-huPON1 was amplified using two terminal primers, 5′-GTTTAACTTT AAGAAGGAGA TATACATATG GCAAAGCTGA CCGC-3′ and 5′-TGAAAATACA GATTCTCACC GCCGGTACCT AATTCACAG-3′, and cloned as described for ΔHDL-huPON1.

 To generate the N-terminal deletion (ΔN) constructs, residues 4 to 17 (LIALTLLGMGLALF) from the leader sequence of PON1 were deleted by PCR amplification of the host genes. The forward primer 5′-AATAATAATC ATATGGCAAA GAGGAACCAC CAGTCTTCTT AC-3′ was used for huPON1 and g2e6p-huPON1; 5′-AATAATAATC ATATGGCGAA AAGGAACCAC CAGTCTTCAG AAC-3′ was used for ΔHDL-huPON1. The reverse primer 5′-AATAATGAAT TCGCCGCCGC TTCCGCTCTG AAAATACAG ATTCTC-3′ was used for huPON1 and ΔHDL-huPON1, and 5′-AATAATAATG GTACCTAATT CACAGTATAA TGCTTTATGG AAAACCG-3′ was used for g2e6p-huPON1. These were cloned as described for ΔHDL-huPON1.

### 2.3. GFP-Fusion Assay for Solubility

 BL21(DE3) cells were transformed with engineered pET11a-PON1-frGFP fusion constructs. LB media (100 mL) supplemented with ampicillin were inoculated with 2 mL of overnight saturated culture grown from a single colony. The cells were grown to OD_600_ *∼*0.7 and induced with 0.1 mM IPTG. The fusion proteins were expressed for 4 h at 30°C and the cells were incubated at 4°C for 6 h before harvesting by centrifugation. Cell pellets were resuspended in PBS and washed with PBS twice before the density of the cells was normalized by adjusting OD_600_ to 0.1. Whole-cell fluorescence was measured in a Perkin Elmer LS50B fluorimeter using 480 nm excitation and 509 nm emission.

### 2.4. Cloning into pHMT

 Genes for full-length and N-terminal deletion variants were cloned into the pHMT vector [30] (kindly provided by Mark Foster, Ohio State Biochemistry) between the NcoI and PstI sites as a C-terminal fusion to maltose-binding protein (MBP). The vector encodes a 6 × His tag at the N-terminus of MBP, a linker (EFGSSRVD), and a TEV protease site (ENLYFQG) between the MBP and fused protein (Figure 2). For the ease of cloning, the SalI site in the original vector was replaced with an NcoI site, and a fragment of unrelated DNA was inserted between NcoI and PstI sites. The huPON1 variant genes were amplified with PCR and cloned between the NcoI and PstI sites.

### 2.5. Cloning into pET11a with a C-Terminal 6 × His Tag

 The MBP fusions of the huPON1 variants were PCR amplified from pHMT using a 5′ primer that removes the N-terminal 6 × His tag and a 3′ primer encoding a new C-terminal 6 × His tag. Genes for the fusions with a TEV protease site between the MBP and protein variants (Figure 2) were cloned into a pET11a vector between the NdeI and XhoI sites.

### 2.6. Fusion Protein Expression and Purification

Origami B (DE3) *E. coli* (Novagen, Madison, WI) were transformed with plasmids encoding the frGFP fusion or MBP fusion constructs and grown overnight to saturation. The MBP fusions were also expressed from cells containing the chaperone plasmid pKJE7 (encoding DnaK, DnaJ, and GrpE, from Takara Bioscience). One liter of LB media supplemented with appropriate antibiotics and 1 mM CaCl_2_ (and 0.1% arabinose when pKJE7 was used) was inoculated with 20 mL of saturated culture and grown at 37°C to an OD_600_ of 0.8. Cells were induced with 0.1 mM IPTG, sometimes after brief incubation in an ice-water bath, and grown at 16–30°C for 4 h to overnight. (Material from different induction and growth conditions had identical specific activity for paraoxon and is presumed to be identical).

 All purification was carried out at 4°C unless stated. Cells harvested by centrifugation at 6,000 g for 10 min were resuspended in lysis buffer (50 mM Tris-HCl, pH 8.0, 50 mM NaCl, 1 mM CaCl_2_, with or without 10% glycerol) supplemented with 1 mM DTT and lysed by extrusion through a needle and sonication. The lysate was incubated with 0.1% Tergitol NP-10 (Sigma-Aldrich) on a nutator at 4°C for 2-3 h. After centrifugation at 27,000 g for 1 h, cleared lysate was mixed with Ni-NTA agarose resin (Qiagen, Valencia, CA) and incubated at 4°C for 3 h with gentle mixing. The slurry was poured into a chromatography column and the flow-through fraction was discarded. The resin was typically washed with lysis buffer containing 25–40 mM imidazole and eluted with lysis buffer containing 150 mM imidazole. It was then exchanged into buffer with 10% glycerol using dialysis or a PD10 desalting column (GE Healthcare) and then into buffer containing 50% glycerol by dialysis. The buffer used was 50 mM Tris-HCl, pH 7.4, 10 mM CaCl_2_ for proteins assayed against paraoxon and phenyl acetate and 50 mM Tris-HCl, pH 8, 50 mM NaCl, 1 mM CaCl_2_, 0.1% Tergitol NP-10, for proteins assayed against EMP and CMP. The difference in buffers was merely due to testing at different times by different researchers. Protein concentrations were determined by Bradford assay (Bio-Rad Laboratories, Hercules, CA) and confirmed by SDS-PAGE. Samples were stored at −20°C.

 For thermal inactivation studies (see below) the huPON1 variants were cleaved away from the MBP fusion protein. After elution from the NiNTA column, proteins were exchanged into a buffer containing 50 mM Tris-HCl, pH 7.4, 10 mM CaCl_2_, 5 mM DTT using a PD10 desalting column. Samples were then treated with TEV protease for 4–6 h at room temperature before subjecting them to Ni-NTA resin binding again. After 4 h of binding at 4°C, resin slurry was poured into a chromatography column, washed with lysis buffer containing 20 mM imidazole, and eluted with lysis buffer containing 150 mM imidazole. Protein samples were exchanged into 50 mM Tris-HCl, pH 8.0, 50 mM NaCl, 1 mM CaCl_2_ using a PD10 desalting column. Samples were further purified over an anion exchange column (Resource Q, GE Healthcare) to separate them from any coeluted proteins. Pure huPON1 and its variants were eluted using a gradient of from 50 mM Tris-HCl pH 8.0, 50 mM NaCl, 1 mM CaCl_2_ to the same buffer with 0.5 M NaCl over 50 mL. Pooled fractions were tested for aryl esterase activity and dialyzed overnight into 50 mM Tris-HCl, pH 8.0, 10 mM CaCl_2_, 50% glycerol, before kinetic measurements.

### 2.7. Construction and Purification of Hum-4E9

 The humanized 4E9 protein (hum-4E9) was designed by introducing the 4E9 mutations (L69G S111T H115W H134R F222S T332S) into the g2e6p-huPON1 sequence. Note that two additional nonpolar-to-polar mutations were made compared to g2e6p-huPON1 (A126T V206T) and that Leu was used at human polymorphic position 55. The amino acid sequence was reverse-translated to retrieve the gene sequence, which was codon-optimized for *E. coli* expression. The gene sequence was ordered from Genewiz, which provided the gene in a pUC57 plasmid. Using BamHI, the hum-4E9 was cloned into pET11a-MBP vector, yielding an MBP-tag at the N-terminus and a 6 × His tag at the C-terminus. The fusion was purified as described above. In order to remove the MBP fusion, the hum-4E9 variant was also cloned into pET32b using NcoI and XhoI. For comparison, we also purified 4E9, as described, from a plasmid kindly provided by Dan Tawfik [15].

### 2.8. Enzyme Kinetics

Kinetic parameters for the hydrolysis of phenyl acetate and paraoxon were determined as described [5] using an assay buffer containing 50 mM Tris-HCl, 10 mM CaCl_2_, pH 7.4. Paraoxon (Sigma) was used from 0.06 to 2.6 mM, phenyl acetate was used from 0.06 to 3.3 mM, and EMP (3-cyano-4-methyl-2-oxo-2*H*-chromen-7-yl ethyl methylphosphonate) and CMP (3-cyano-4-methyl-2-oxo-2*H*-chromen-7-yl cyclohexyl methylphosphonate) were used from 0.005 mM to 0.5 mM. The initial rate of formation of hydrolysis product at 25°C was monitored by following the absorbance at 405 nm for *p*-nitrophenolate from paraoxon (*ε* = 14,320 M^−1^ cm^−1^), at 270 nm for phenol from phenyl acetate (*ε* = 1,310 M^−1^ cm^−1^), and at 405 nm for 7-hydroxy-4-methyl-3-cyanocoumarin (MeCyC, *ε* = 37,000 M^−1^ cm^−1^) from EMP and CMP, using an Agilent 8453 UV-Vis spectrophotometer or a SpectraMax M5 Pro multiwell plate reader (Molecular Devices, Sunnyvale, CA) and Greiner One UV Star plates. Stocks of phenyl acetate, paraoxon, EMP, and CMP were prepared in methanol. Kinetic parameters for EMP and CMP were determined at 2% constant methanol. Parameters were derived by fitting a Michaelis-Menten model of steady-state enzyme kinetics to the data with KaleidaGraph (Synergy Software, Reading, PA). EMP and CMP were kindly provided by Yacov Ashani, Weizmann Institute of Science, and were synthesized in house by the method of Ashani et al. [31] (S. Muthukrishanan, D. Mata, TJM and C. Hadad, unpublished).

 To test activity against the more toxic *S_P_* isomer of CMP, the protocol from Gupta et al. [15] was followed as described. In short, racemic CMP (0.025 mM) in the presence of 3B3 PON1 (provided by Dan Tawfik) was incubated in 50 mM Tris-HCl, 10 mM CaCl_2_, pH 7.4 for 30 min at 4°C to deplete the *R_P_*-isomer from the reaction. The reaction mixture (150 *μ*L) and 50 *μ*L of diluted enzyme were added to a 96-well plate.

To determine the kinetics of cyclosarin (GF) hydrolysis, racemic cyclosarin was obtained from the US Army Edgewood Chemical Biological Center (Aberdeen Proving Ground, MD). Analysis by NMR spectroscopy showed it to be >95% pure. Stock solutions of GF in saline were prepared at 2 mg mL^−1^ and stored at −70°C. Enzyme was incubated with 0.3125 mM GF in 10 mM MOPS, 2 mM CaCl_2_ at room temperature. At specific time intervals, 100 *μ*L aliquots were removed and inactivated through extraction with an equal volume of ethyl acetate containing 50 *μ*M diisopropyl fluorophosphate (DFP; internal standard); this extraction both inactivates the enzyme and prevents racemization of nonhydrolyzed GF stereoisomers. The organic layer (containing nonhydrolyzed GF) was then removed and analyzed by gas chromatography/mass spectrometry (GC/MS).

 Chiral gas chromatographic analysis of GF was performed using an Agilent 7890 gas chromatograph (Foster City, CA) fitted with a 20 m × 0.25 mm internal diameter ASTEC G-TA column (Astec, Whippany, NJ). Helium was used as the carrier gas at an average linear velocity of 54.5 cm s^−1^. The oven temperature was held initially at 70°C for 1 min and then ramped from 70 to 160°C at a rate of 10°C min^−1^. Split injections (50 : 1) of 1 uL volume were made using an Agilent 7693 autosampler. The injection port temperature was 210°C and the split vent delay was set at 1 min. The GC was interfaced to an Agilent 5975 mass spectrometer (MS) with an electron impact ion source. The MS operating conditions were as follows: ion source pressure approximately 1.0 × 10^−5^ Torr; source temperature, 230°C; quadrupole temperature, 150°C; electron energy, 70 eV; transfer line temperature, 265°C. The MS was operated using selected ion monitoring. Ion pairs m/z 99 and 67 and m/z 101 and 127 were monitored for GF and DFP, respectively. A dwell time of 100 ms for each ion pair resulted in a scan rate of 8.26 cycles s^−1^. Rate constants (*k_app_*) for hydrolysis of racemic GF were derived by using nonlinear regression to fit hydrolysis progress curves to a single-phase decay model using Prism 4.03 (GraphPad Software, La Jolla, CA). Relative stereoisomeric preference was calculated by determining the ratio of the rates of hydrolysis of each enantiomer of GF.

### 2.9. Thermal Inactivation and Residual Activity Determination

 Protein samples were heated for 10 min at different temperatures ranging from 25°C to 80°C. After a brief incubation on ice and centrifugation in a picofuge at 2,200 g for 2 min, their activities were determined from EMP (0.35 mM) or phenyl acetate (3.6 mM) hydrolysis. The residual hydrolysis activity from incubation at 20°C (phenyl acetate) or 25°C (EMP) was taken as 100%.

## 3. Results

### 3.1. Design of Rationally Engineered Variants

 Based on what is known about the leader sequence, the crystal structure of G2E6, and sequence comparison of G2E6 to huPON1, we designed three variants of huPON1 to examine their effects on the solubility of the protein. The first variant, ΔN-huPON1, is a deletion of residues 4–17, which includes most of the leader sequence. The first residue resolved in the crystal structure of G2E6 (PDB ID: 1V04 [16]) is Leu16. The first three residues, MAK, are fairly soluble and the small size of Ala2 likely contributes to homogeneous demethioninylation in *E. coli*. Residues 16 and 17 are Leu and Phe, so the 4–17 deletion results in removal of basically all of the N-terminal hydrophobic residues.

 Our second hypothesis was that increasing the polar character of the putative HDL binding site, which is defined by a large number of surface hydrophobic residues, would increase the solubility of the protein. We speculated that this might not affect the structure or activity of the protein significantly since it is on the surface and pointed away from the active site. Residues proposed to be involved in HDL anchoring lie principally in Helix 2 and the adjacent loops, as well as Helix 1 [16] (Figure 3). We modified this surface based on inspection of the crystal structure of G2E6 with a limited library of polar amino acids (Glu, Gln, and Lys) to yield ΔHDL-huPON1 (Y24E Y185E F186Q L187K Y190K L191Q W194K L198E L200Q W202K M289Q F293E).

 Our third hypothesis was that only a subset of the surface changes in the directed evolution of G2E6 was responsible for increasing the solubility of the protein. In particular, we speculated that sites that became significantly more polar (either nonpolar to polar or charged, or polar to charged) would contribute to most of the solubility increase seen with G2E6 [17]. We chose 15 sites where residues became significantly more polar from huPON1 to G2E6 (I5T N19R Q21K L31H N78D N80D S81K P82S L98S G101E A137S Q192K Y197H N265D N309D). We then examined whether any of the mutations were in the vicinity of any of the other 44 mutations present in G2E6, and we found a single case wherein Lys192 made a hydrogen bond to Ser166. Position 192 is an interesting site because it is a site of human polymorphism, where Gln and Arg are common [32]. Also, of all 59 mutations in G2E6, only five positions are within 9 Å of the active-site His115 residue, and of those only the side chains of Ser166 and Lys192 point toward the active site. Consequently, with the idea that coupling between these two positions could be important, we also included the N166S mutation to yield g2e6p-huPON1.

 We also engineered several combinations of these variants, by combining the N-terminal deletion to ΔHDL-huPON1 (to yield ΔN-ΔHDL-huPON1) and the g2e6p-huPON1 (to yield ΔN-g2e6p-huPON1), to examine the potential for additivity in increasing solubility.

### 3.2. GFP-Fusion Screen for Solubility

 To assess the solubility of these engineered variants, we turned to the GFP-fusion screen developed by Waldo and colleagues [24]. In this screen, a protein of interest is fused to the N-terminus of “folding reporter” GFP, and cellular fluorescence develops in proportion to the solubility of the POI. In reality, the cellular fluorescence is related to the amount of soluble protein, but in this case all of the tested variants of PON1 expressed in similar significant quantities in whole-cell lysate (not shown), so we can assume that increased fluorescence is due to increased partitioning into the soluble fraction. We constructed frGFP by combining EGFP (F64L S65T) [27] with Stemmer's “cycle 3” GFPuv (F99S M153T V163A) [26], and we generated our own fusion construct in a pET11a plasmid. We also tested the screen by assaying unfused frGFP as well as fusions of T4 lysozyme, yeast triosephosphate isomerase (TIM), G2E6 PON1, and human PON1. Cells with the T4 lysozyme fusion were more fluorescent than those with the unfused GFP; yeast TIM resulted in comparable fluorescence, G2E6 with lower fluorescence, and huPON1 with even lower fluorescence than G2E6 (data not shown). Cells were washed with phosphate-buffered saline and normalized for cell density before fluorescence was measured.

**Figure 4:**
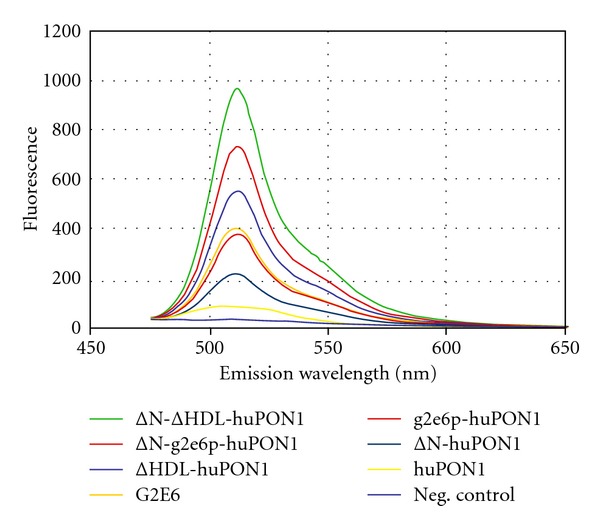
*GFP-fusion screening for solubility.* The fluorescence level is shown for normalized numbers of cells in PBS with frGFP fusions of the PON1 variants. Results from multiple trials typically varied by less than 10%. Negative control cells contained no frGFP fusion. For clarity, the constructs are listed in the legend in the same order from top to bottom as the maxima of the emission spectra.

 The solubility results are shown in Figure 4. As we observed in our controls, the fluorescence was about 4.5-fold greater for G2E6 than huPON1. The three engineered variants were all more soluble than huPON1: the fluorescence for ΔN-huPON1 was about twice that of huPON1, g2e6p-huPON1 was about four times as fluorescent, and ΔHDL-huPON1 was about six times as fluorescent. Interestingly, g2e6p-huPON1 had nearly the same fluorescence level as G2E6, consistent with our hypothesis that the increased solubility of G2E6 was essentially entirely due to the subset of residues that became more polar on the surface of PON1. Mutation of the hydrophobic residues in the putative HDL binding site to polar and charged residues (ΔHDL-huPON1) had the greatest effect on the protein solubility, exceeding that of G2E6 by almost 50%.

 The removal of the hydrophobic N-terminal leader had the least effect of the three solubilizing concepts (*∼*2.4-fold). However, the effects of the N-terminal deletion were mostly additive with the ΔHDL and g2e6p mutation sets. The fluorescence increased an additional 2-fold for ΔN-g2e6p-huPON1 over g2e6p-huPON1 and an additional ~1.8-fold for ΔN-ΔHDL-huPON1 over ΔHDL-huPON1.

### 3.3. Expression and Purification of Engineered Variants

**Figure 5:**
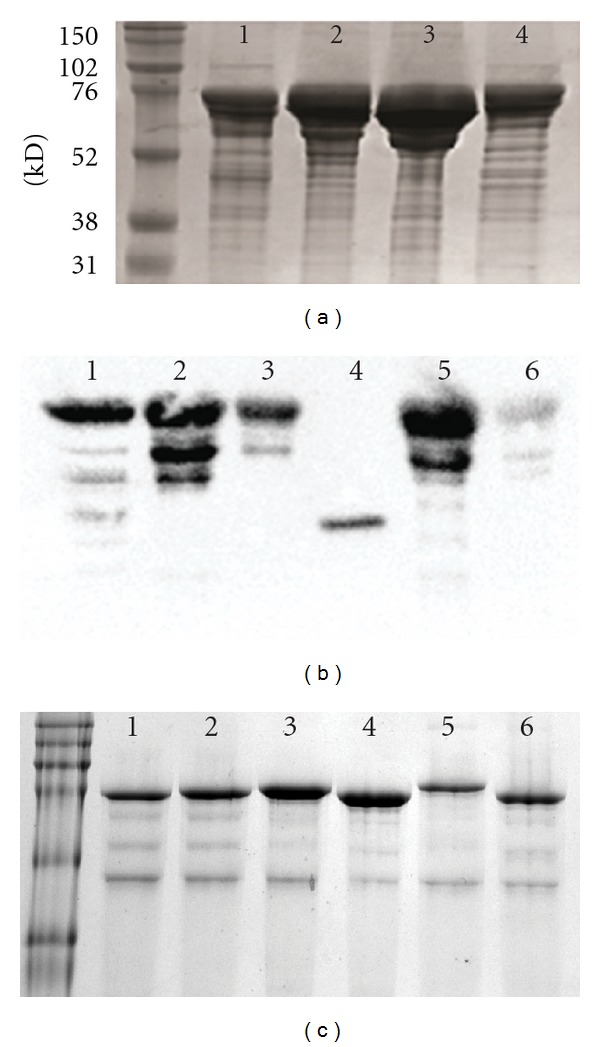
*Purification of huPON1 variants from E. coli*. (a) SDS-PAGE of purification from His_6_-MBP-PON1 fusions from the pHMT plasmid using NiNTA chromatography. (1) ΔN-huPON1; (2) ΔHDL-huPON1; (3) ΔN-ΔHDL-huPON1; (4) g2e6p-huPON1. (b) Blot of SDS-PAGE of PON1 variants as His_6_-MBP-PON1 fusions with HisProbe-HRP (Pierce), demonstrating that many of the smaller proteins bear the 6 × His tag and are likely proteolytic fragments. (1) ΔHDL-huPON1 lysate; (2) and (3), purified ΔHDL-huPON1; (4) cleaved MBP; (5) purified g2e6p-huPON1; (6) purified ΔN-huPON1. (c) SDS-PAGE of purified proteins from the MBP-PON1-His_6_ constructs in pET11a with coexpression of DnaK/DnaJ/GrpE from pKJE7 (Takara Bioscience). (1) huPON1; (2) ΔN-huPON1; (3) ΔHDL-huPON1; (4) ΔN-ΔHDL-huPON1; (5) g2e6p-huPON1; (6) ΔN-g2e6p-huPON1.

 We wished to purify the huPON1 solubilized variants to measure their activity and stability, as well as to verify their soluble expression. However, we found that the frGFP fusions were expressed at such low levels that it was inconvenient to work with them. As a result, we recloned the constructs, fusing a hexahistidine tag and maltose-binding protein to the N-terminus of the huPON1 variants. When these variants were purified by NiNTA affinity chromatography, they appeared to copurify with a significant number of smaller proteins at reduced but significant levels (Figure 5). Note that, for the MBP fusions, the amount of soluble protein captured in the purification was greatest for ΔN-ΔHDL-huPON1 and then ΔHDL-huPON1; g2e6p-huPON1 and ΔN-huPON1 were purified in comparable lower amounts, consistent with the screening data. It proved difficult to purify the proteins with increasingly stringent washes, which made us suspect that the other bands on the SDS-PAGE gel were truncation products of the full-length constructs. A blot with the anti-His_6_ reagent HisProbe-HRP confirmed that the smaller proteins contained a hexahistidine tag.

 These truncation products likely arose from proteolysis due to poor protein stability or inefficient folding of the huPON1 variants. We speculated that moving the 6 × His tag to the C-terminus might allow us to capture the full-length proteins. Consequently, we recloned selected constructs into a pET11a vector as fusions with MBP on the N-terminus and a hexahistidine tag on the C-terminus. However, the yields of these full-length proteins were low. We previously observed a similar result with huPON1 and found that coexpression with DnaK/DnaJ/GrpE chaperones as well as expression at lower temperatures enhanced the production of full-length protein (V. Shete, B. Competty, TJM, manuscript in preparation). When the MBP-PON1-His_6_ constructs were expressed in *E. coli* overexpressing the DnaK chaperone system, full-length huPON1 variants could be purified at higher yields with no significant evidence of truncation (Figure 5). A protein the same size as DnaJ was found to copurify with the MBP fusions. We verified that no significant hydrolytic activity above background could be observed from the lysates of cells overexpressing the DnaK chaperones.

 Because G2E6 can be purified as a thioredoxin or MBP fusion (not shown) with no evidence of these truncation products, it is likely that all of the huPON1 derivatives fold less well than G2E6.

### 3.4. Activity and Stability of Engineered Variants

**Figure 6:**
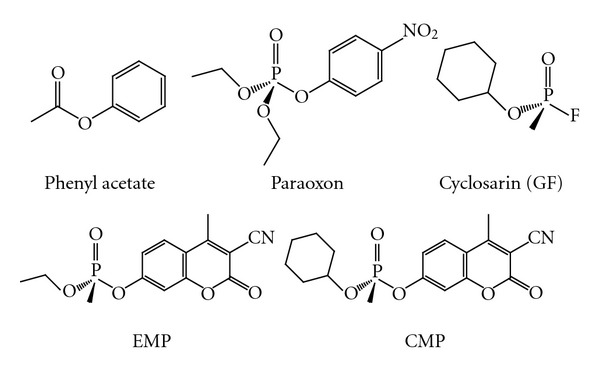
*Structures of the substrates used in this study.* CMP, EMP, and GF are shown as the *S_P_* enantiomorphs.

 The activity of our engineered PON1 variants against phenyl acetate and the OP compounds paraoxon and EMP was determined and compared with huPON1 and G2E6 (see Figure 6 for structures of the substrates). Because the activity levels were modest and initial trials suggested that the *K_m_* values were likely to be near the top of the concentration range that could be tested with each substrate, specific activities are reported for some substrates (Table 1).

 Assuming that 3.3 mM phenyl acetate nearly saturates our PON1 variants, we can calculate a comparable specific activity for huPON1 based on the *k_cat_* value we reported previously [5]. The huPON1 used in that study was not an MBP fusion, but if we calculate the specific activity using the mass of the fusion for comparison, we arrive at a specific activity of 690 *μ*mol min^−1^ mg^−1^. The corresponding value for G2E6 is 820 *μ*mol min^−1^ mg^−1^. When we expressed huPON1 as an MBP fusion exactly as we did for the huPON1 variants here, the specific activity was 4-fold lower than that reported previously for enzyme purified from 293T cells. All of the variants engineered here had considerably lower activity. The ΔN-huPON1 and g2e6p-huPON1 were about 10-fold lower in activity than huPON1. The ΔHDL-huPON1 variant was about another 20-fold lower than the other two variants. Moreover, the N-terminal deletion reduced the activity of g2e6p-huPON1 by 5-fold, and it approximately halved the already-low activity of the ΔHDL-huPON1. While all three of these methods of solubilization did in fact produce more soluble material, the protein produced had lower specific activity, with the most significant reduction in activity for the ΔHDL-huPON1.

 The activity of huPON1 against paraoxon is considerably lower than against phenyl acetate (the corresponding specific activity is 0.3 *μ*mol min^−1^ mg^−1^), so it is not surprising that we were only able to detect activity against paraoxon with a single variant, the g2e6p-huPON1. The g3e6p-huPON1 activity was about 30-fold below the calculated specific activity for huPON1 expressed in 293T cells, on par with the corresponding reduction in phenyl acetate activity. However, when huPON1 was expressed the same way, the activity against paraoxon was actually 35-fold lower than g2e6p-huPON1 and about 100-fold lower than the 293T-expressed material. The reason for this reduction against paraoxon is not clear. Using EMP, which is an excellent OP substrate for PON1, we also observed that g2e6p-huPON1 was the most active variant, and it was about 2.5-fold more active than ΔN-huPON1 and 100-fold more active than ΔN-g2e6p-huPON1. We could not detect activity against EMP with ΔHDL-huPON1. The activity of g2e6p-huPON1 was also slightly higher than huPON1 with EMP (*∼*1.5-fold), although much less so than with paraoxon.

**Figure 7:**
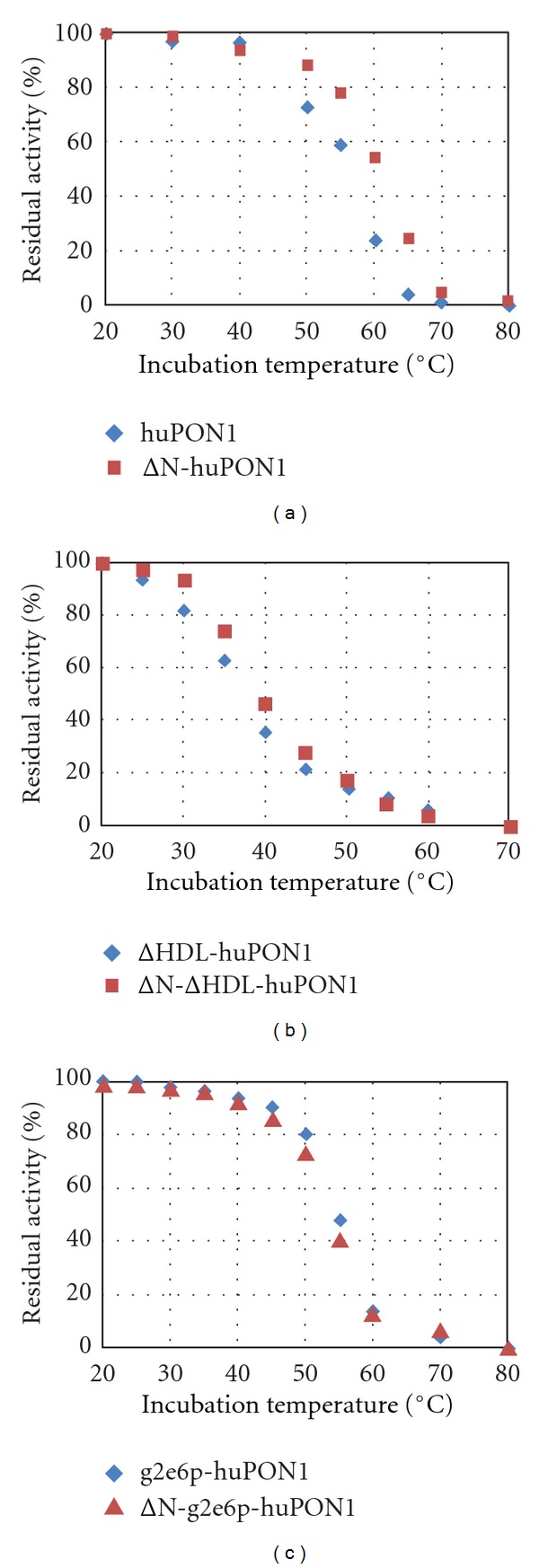
*Stability of huPON1 variants.* The residual activities against phenyl acetate after 10 min of incubation at the indicated temperatures are shown. The residual activity after incubation at 20°C was taken as 100%. (a) huPON1 and ΔN-huPON1; (b) ΔHDL-huPON1 and ΔN-ΔHDL-huPON1; (c) g2e6p-huPON1 and ΔN-g2e6p-huPON1.

 We were also interested in the effects of each of these solubilizing sets of mutations on the stabilities of the resulting proteins. Because PON1 denatures irreversibly upon heating, thermal inactivation is a good measure of the relative stability of the variants [33]. With phenyl acetate activity as the read-out, the *T*_1/2_ for huPON1 was roughly 55–60°C (Figure 7). The ΔN-huPON1 was increased slightly in stability, to about 60°C. Both g2e6p-huPON1 and ΔN-g2e6p-huPON1 have *T*_1/2_ values close to 55°C, and both ΔHDL-huPON1 and ΔN-ΔHDL-huPON1 have *T*_1/2_ values close to 40°C. A similar experiment using EMP as the substrate produced similar results, with huPON1 showing a midpoint in the inactivation curve around 55°C and ΔN-huPON1 and g2e6p-huPON around 50°C. Overall we conclude that the N-terminal deletion had little effect on the stability, the g2e6p mutations reduced the stability of huPON1 slightly, and the ΔHDL mutations reduced it significantly.

### 3.5. Humanization of 4E9

**Figure 8:**
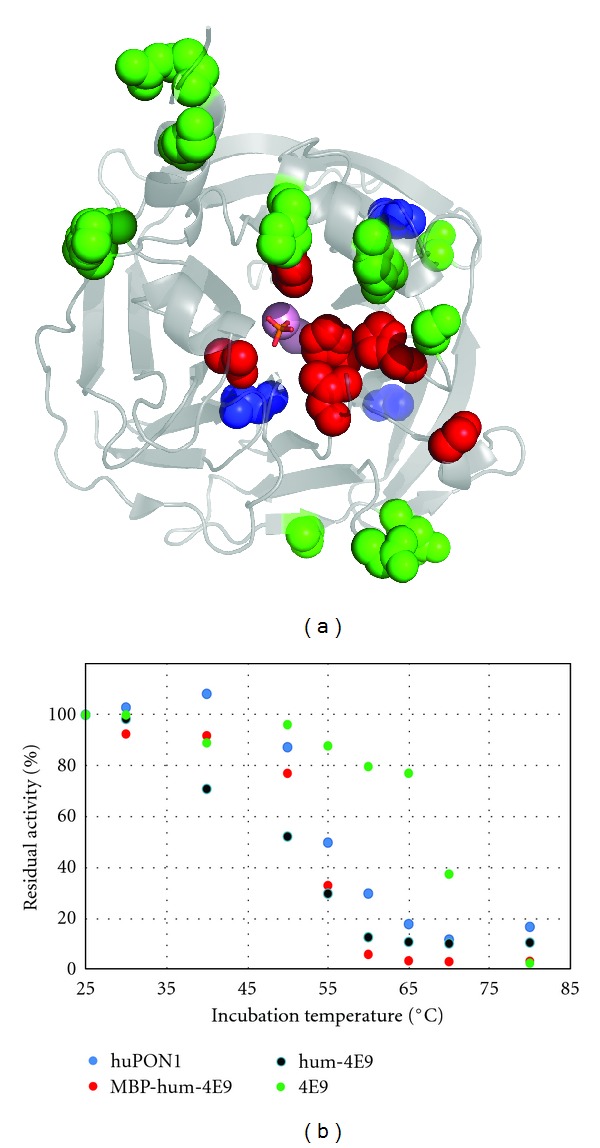
*Humanization of 4E9.* (a) The g2e6p mutations are shown in green spheres; the three additional mutations M55L, A126T, and V206T, are shown in blue spheres; the 4E9 mutations (with respect to G3C9) are shown in red spheres. (b) Thermal inactivation of hum-4E9 produced with and without MBP fusion is compared to huPON1 and 4E9. Residual activity here was from EMP hydrolysis rather than phenyl acetate because the H115W mutation renders PON1 inactive against phenyl acetate.

We wished to test whether we could produce a solubilized huPON1 with significant activity toward an OP compound, effectively “humanizing” an engineered PON1. The g2e6p-huPON1 variant appeared to afford the best combination of solubility, activity, and stability from among our original variants, so we elected to use those mutations to humanize an engineered PON1. Gupta and colleagues recently reported the engineering of a PON1 variant called 4E9 that has significant activity toward the cyclosarin (GF) analog CMP and notably increased activity against the more toxic *S_P_* enantiomorph of CMP [15]. It also has significant activity against authentic GF, as determined from an AChE inactivation assay in which GF is generated *in situ* at low concentrations. 4E9 is derived from G3C9, with the mutations L69G S111T H115W H134R F222S T332S, all of which are in the presumed active site except for S111T. Therefore, we generated a variant of huPON1 with surface solubilizing mutations derived from G2E6 (akin to the g2e6p-huPON1 described above and in Figure 1) and the 4E9 mutations obtained during directed evolution. The resulting variant, hum-4E9 (Figure 8), has two additional nonpolar-to-polar mutations that we did not elect to make in the g2e6p-huPON1: A126T and V206T. As an additional test of the solubilization afforded by the surface polar mutations, we not only expressed hum-4E9 as an MBP fusion with C-terminal 6 × his tag, but we also produced it with no fusion partner (with a C-terminal 6 × His tag only, which is also present in G3C9 and 4E9). The MBP fusion of hum-4E9 was purified in similar yield to the other MBP fusions of the huPON1 variants and G2E6. The unfused hum-4E9 was produced in about 5-fold lower yield than this and approximately 25-fold lower yield than 4E9 itself.

### 3.6. Characterization of Hum-4E9

 Not only was hum-4E9 expressed in significant quantities both with and without an MBP fusion tag, but the enzyme was also very active (Table 2). The *k_cat_*/*K_m_* for hydrolysis of EMP was 6,800 M^−1^ s^−1^ for the MBP fusion protein and surprisingly was slightly higher (7,500 M^−1^ s^−1^) for the protein produced without the fusion partner. This represents more than a 20-fold increase over g2e6p-huPON1. We have observed that the activity of G3C9 and 4E9 against EMP is similar (within 2-fold, CKH and TJM, unpublished), suggesting that the increase in EMP activity between g2e6p-huPON1 and hum-4E9 cannot be attributed entirely to the active-site mutations.

 The rates of hydrolysis of CMP were also high. For this substrate, the MBP fusion protein had slightly higher activity than the untagged version (6,000 M^−1^ s^−1^ versus 3,200 M^−1^ s^−1^). The value for the MBP fusion is within 3-fold of the CMP activity of 4E9 measured under identical conditions. There is also significant activity against authentic GF (~6,000 M^−1^ s^−1^). Tawfik reports that G3C9 has no detectable activity against the *S_P_* isomer of CMP, and we have also observed this (CKH and TJM, unpublished). In the *R_P_*-CMP depletion assay, we found that 4E9 and hum-4E9 had the same level of activity against *S_P_*-CMP, despite the overall lower level of activity for hum-4E9 against CMP. The ratio of the rate constants for hydrolysis of the *R_P_* : *S_P_* isomers of GF was about 4 : 1, suggesting that hum-4E9 displays substantial activity against the more toxic isomer of GF, even though it is still selective for the less toxic isomer.

 We also examined the stability of hum-4E9 by thermal inactivation, using EMP as a substrate (Figure 8). The apparent *T*_1/2_ values were *∼*50°C for both the MBP fusion and the unfused protein. This level of stability is consistent with that of g2e6p-huPON1.

## 4. Discussion

 There is relatively little known about the effects of mutations on protein solubility or about how to engineer proteins for increased solubility. This is one reason that Waldo and colleagues invented their GFP-fusion screen, so that directed evolution from random mutagenesis could be used to select for more soluble proteins [24]. It seems intuitively reasonable that increasing the fraction of polar and charged residues on the surface of a protein would increase solubility, but natural proteins generally have the same fraction of polar residues on their surfaces as they do in overall composition, meaning that about half of surface residues are hydrophobic. Mutations to the surfaces of proteins are often naively thought of as neutral, but solubilizing mutations also affect the solubility of the unfolded state. Consequently, they can affect both the stability and folding of proteins. Proteins from thermophiles differ more from their mesophilic counterparts on their surfaces than in factors such as hydrophobic core packing [34].

 Here we explored the effects of three different kinds of mutations to PON1, to assess their impact on solubility, activity, and stability. Two of the approaches were rational—we removed the hydrophobic signal sequence from the N-terminus of the proteins, and we solubilized the putative HDL binding site by replacing a large number of closely grouped surface hydrophobic residues with polar and charged amino acids. The third approach was based on the directed evolution of G2E6. We postulated that most of the increased solubility of G2E6 over huPON1 arose from substitution of residues on the surface to more polar amino acids. We constructed the ΔHDL and g2e6p mutants of huPON1, as well as the ΔN mutants of huPON1 and the other two variants. We examined the solubility of the variants using GFP-fusion screening. These fusions were produced at low levels, so we recloned the variants as MBP fusions for higher yield expression and *in vitro* characterization.

 All three of the sets of mutations increased the solubility relative to huPON1, but to different degrees. The deletion of the N-terminal signal sequences had the least effect and afforded roughly the same amount of solubilization to the other variants as it did to huPON1. These results suggest that the effects of the different sets of solubilizing mutations can be at least partially additive. The N-terminal deletion also resulted in only a modest decrease in the activity and little change in the stability of huPON1. It is interesting that there is any decrease in activity for this variant (ΔN-huPON1), since the N-terminus is disordered in the crystal and not near the active site of the protein. The ΔHDL mutant afforded the greatest solubilization, but at the highest cost to both activity and stability. While these mutations were a much more radical change to the protein than the N-terminal deletion, it is still somewhat surprising that they have such a profound impact on the activity of the protein. It is known that the activity of PON1, particularly lactonase activity, is stimulated by binding to HDL [20]. While there is no HDL in these preps, either remnants of bacterial cellular lipids (such as lipopolysaccharide) or detergent added to the purification may partially substitute for HDL. It is possible that binding to lipid has either a direct effect on the structure of the active site or that it affects other properties such as enzyme dynamics that cannot be easily recapitulated in mutants that do not bind lipid.

 Perhaps the single most important result of this work is the demonstration that the subset of surface mutations that become more polar in G2E6 entirely account for the increased solubility of G2E6 over huPON1. This is potentially a useful approach for minimizing the number of mutations that arise from random mutagenesis and directed evolution of other proteins. The g2e6p-huPON1 variant was only slightly destabilized relative to huPON1, and it was reduced in activity by only a small amount. Mutations from huPON1 to G2E6 mostly arose from residues that are found in other mammalian paraoxonases, which likely aided in making them minimally detrimental to the structure and function. Still, we can conclude that some of the other 43 mutations from huPON1 to G2E6 must be important for other parameters, such as the higher activity of G2E6 relative to g2e6p-huPON1.

 For huPON1 and all of the soluble variants, we observed that considerable amounts of proteolytic products were purified and that these could be eliminated by coexpression of the DnaK chaperone system. Such products are not observed with thioredoxin or MBP fusions of G2E6, which suggests that one of the major effects of the directed evolution to G2E6 was an increase in the folding rate of the protein. Since the mutations that solubilized the g2e6p-huPON1 were alone not sufficient to eliminate these products, we can conclude that this effect also arises from some of the other 43 residues that change from huPON1 to G2E6. It will be interesting to determine which mutations are responsible for this effect. Overall, our results emphasize that surface mutations, aside from having solubility effects, are often far from neutral on the stability and folding of proteins.

 The idea of “humanization” of engineered proteins was conceived in the development of antibody drugs, where it is necessary to raise antibodies in mice or other animals, but a consequence of which is immune response to the constant regions of the heterologous antibodies. It is often possible to engineer molecules with human antibody surfaces but affinity matured binding sites, albeit sometimes with loss of affinity or stability [25]. There are not yet sufficient data to know if mammalian chimeric PON1 variants like G3C9 and its progeny will elicit immune responses, but it is a concern given the degree of sequence divergence. Using an approach reminiscent of antibody humanization, we implanted the active site of the engineered 4E9 variant into huPON1 and solubilized huPON1 with the surface polar mutations from G2E6. We have shown the activity and the effects of mutations in the huPON1 and G2E6 backgrounds to be highly divergent, so it was not clear that introduction of the 4E9 active-site mutations would afford high CMP and GF activity as observed for 4E9. Interestingly, the engineered variant does exhibit 4E9-like activity, including similar stereochemical preferences for CMP and GF. Our hum-4E9 still differs in a significant number of positions from huPON1 and expresses at lower levels than 4E9, so further engineering may be required to afford an ideal molecule. Nonetheless, the current data present a promising proof of principle that protein solubility can be altered in a controlled and rational way.

 Finally, it is of note that two of the mechanisms that we chose to solubilize huPON1 (ΔN and ΔHDL) are likely to reduce or exclude HDL binding, while the g2e6p mechanism is likely to be compatible with it. These mutants, and perhaps related mutants in G3C9, may be useful for examining the role of HDL binding in the function and regulation of PON1. For example, we have found that mutation of a single Trp in the putative HDL binding site of G3C9 dramatically increases the solubility of G3C9 and consequently may reduce its binding to HDL (R. Baldauff and TJM, unpublished). It is unclear what effect untethering PON1 from HDL will have on its physiological function, its serum lifetime, or its recognition by the immune system, but it may help solve the puzzle of exactly what PON1 is doing *in vivo*.

## Figures and Tables

**Table 1:** *Aryl esterase and OPase activity of huPON1 variants.* Phenyl acetate and paraoxon specific activities were determined at 3.3 mM and 2.6 mM substrate, respectively. Errors for the specific activities are the standard errors from 3 trials. EMP values were determined at constant 2% methanol, and the errors are with respect to the fit. *n.d.*: not detected. —: *k_cat_*/*K_m_* is reported from a linear fit of initial rate versus substrate concentration when the enzyme could not be saturated under experimental conditions.

Variant	Phenyl acetate	Paraoxon		EMP	
	specific activity *μ*mol min^−1^ mg^−1^	specific activity *μ*mol min^−1^ mg^−1^	*k_cat_* s^−1^	*K_m_* mM	*k_cat_/K_m_* M^−1^ s^−1^
huPON1	160 ± 20	0.003 ± 0.001	0.027 ± 0.001	0.14 ± 0.01	190 ± 20
ΔN-	17 ± 9	*n.d.*	—	—	117 ± 8
g2e6p-	20 ± 5	0.011 ± 0.002	0.041 ± 0.003	0.14 ± 0.02	300 ± 50
ΔN-g2e6p-	4 ± 1	*n.d.*	—	—	2.2 ± 0.1
ΔHDL-	1.2 ± 0.2	*n.d.*		*n.d.*	
ΔN-ΔHDL-	0.8 ± 0.2	*n.d.*		*n.d.*	

**Table 2:** *Activity of hum-4E9. k_cat_*/*K_m_* values were determined under constant 2% methanol conditions for all three substrates. None of the enzymes could be saturated with those substrates under experimental conditions. *S_P_*-CMP specific activities were determined at *∼*0.0125 mM *S_P_*-CMP. GF *k_app_* hydrolysis rate constants and stereoisomeric preferences were determined using GF at 0.315 mM. ND: not determined.

	paraoxon	EMP	CMP	*S_P_*-CMP	GF	
Variant	*k_cat_*/*K_m_* M^−1^ s^−1^	*k_cat_*/*K_m_* M^−1^ s^−1^	*k_cat_*/*K_m_* M^−1^ s^−1^	specific activity nmol min^−1^ mg^−1^	*k_app_* M^−1^ s^−1^	*R_P_* : *S_P_* ratio
4E9	3, 100 ± 200	24, 000 ± 1, 000	17, 000 ± 3, 000	1.1 ± 0.1	ND	ND
hum-4E9	750 ± 20	7, 500 ± 600	3, 200 ± 300	1.10 ± 0.05	8,700	3.5 : 1
MBP-hum-4E9	230 ± 80	6, 800 ± 300	6, 000 ± 2000	ND	5,400	6 : 1
